# An Enhanced Source Location Privacy based on Data Dissemination in Wireless Sensor Networks (DeLP)

**DOI:** 10.3390/s19092050

**Published:** 2019-05-02

**Authors:** Naveed Jan, Ali H. Al-Bayatti, Naseer Alalwan, Ahmed Ibrahim Alzahrani

**Affiliations:** 1 Department of Electrical Engineering, CECOS University of IT and Emerging Sciences, Peshawar 250000, Pakistan; 2 School of Computer Science and Informatics, De Montfort University, Leicester LE1 9BH, UK; alihmohd@dmu.ac.uk; 3 Department of Computer Science, Community College, King Saud University, Riyadh 11437, Saudi Arabia; nalalwan@ksu.edu.sa (N.A.); ahmed@ksu.edu.sa (A.I.A.)

**Keywords:** wireless sensor network, contextual privacy, source location privacy

## Abstract

Wireless Sensor Network is a network of large number of nodes with limited power and computational capabilities. It has the potential of event monitoring in unattended locations where there is a chance of unauthorized access. The work that is presented here identifies and addresses the problem of eavesdropping in the exposed environment of the sensor network, which makes it easy for the adversary to trace the packets to find the originator source node, hence compromising the contextual privacy. Our scheme provides an enhanced three-level security system for source location privacy. The base station is at the center of square grid of four quadrants and it is surrounded by a ring of flooding nodes, which act as a first step in confusing the adversary. The fake node is deployed in the opposite quadrant of actual source and start reporting base station. The selection of phantom node using our algorithm in another quadrant provides the third level of confusion. The results show that Dissemination in Wireless Sensor Networks (DeLP) has reduced the energy utilization by 50% percent, increased the safety period by 26%, while providing a six times more packet delivery ratio along with a further 15% decrease in the packet delivery delay as compared to the tree-based scheme. It also provides 334% more safety period than the phantom routing, while it lags behind in other parameters due to the simplicity of phantom scheme. This work illustrates the privacy protection of the source node and the designed procedure may be useful in designing more robust algorithms for location privacy.

## 1. Introduction

A network is composed of a set of vertices and a set of edges that connect these vertices. The air interface provides the facility of edges in wireless networks. Wireless Sensor Network (WSN) is a network of sensor nodes that are distributed in an ad-hoc manner. The sensors are designed and programmed to sense some physical phenomenon, process it, and then report it to the Base Station (BS). The sensors are normally deployed in a vast area and every sensor may not directly communicate with the base station. The information is conveyed from source node to destination using multi hop routing [1].

**Figure 1:**
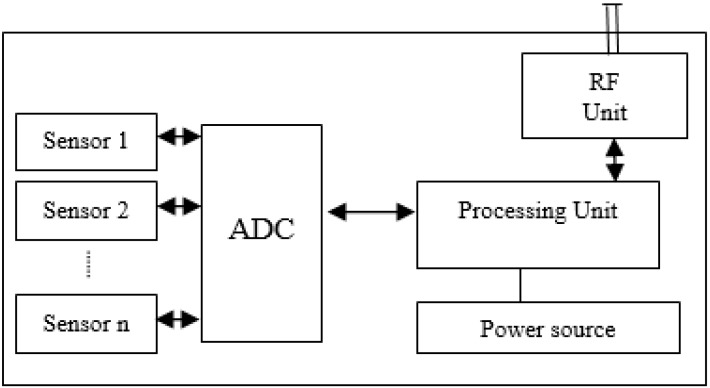
Block diagram of a simple Mote.

These wireless sensor nodes (motes) are considered as the basic building blocks of a wireless sensor network and they have very limited resources in terms of memory, communication range, and computational power. A mote consists of the basic parts: (1) Processing unit; (2) Radio Frequency (RF) transceiver unit to communicate with outside world; (3) Power source; and, (4) One or more sensors to detect physical world, as shown in Figure 1.

### 1.1. Motivation

WSN can be deployed for many applications from military tracking to habitat monitoring of animals [2,3,4,5,6,7,8,9]. The un-attended nature of WSN, like the one discussed in the habitat monitoring of animals in large geographical areas, always contain a threat to privacy issues. The adversaries can easily gain access to the wireless sensor network because of the open architecture of the underlying sensor technology [10]. Intelligent Spectrum analyzers, along with antennae, memory, and processing devices may be used for eavesdropping in the deployed network and privacy may be compromised [11]. Privacy is a guarantee that information that is gathered might be only observed or deciphered by the authorized parties. The monitoring application can be misused by the attacker to hunt the animals, hence severely affecting the aim of the monitoring application Location privacy is not just limited to remote WSN, but also information leakage in the location-based services by third party observers [12] and node location privacy in the industrial wireless sensor network [13] is also a big threat to privacy issues.

### 1.2. Problem Statement

**Figure 2:**
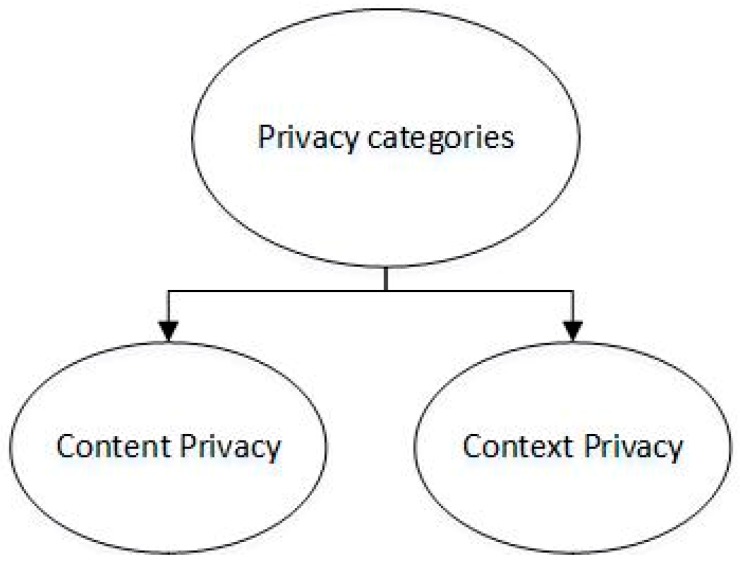
Privacy categories in wireless sensor networks.

Source location privacy (SLP) is an important issue and a lot of research work has been carried regarding it. A number of attacks possible on the wireless sensor networks are discussed in [14]. There are two types of privacy classes: content-oriented privacy and contextual privacy. Content oriented privacy ensures that the contents of the packets are not exposed or modified while traversing from source to sink. This privacy can be ensured with sophisticated encryption algorithms and is mostly addressed by recent research carried out by technical community [15,16,17], and it is not the scope of this paper. Contextual privacy is concerned with protecting the context that is associated with the sensed data in course of measurement and transmission. This may endanger the privacy of the asset if the sensor network is deployed for its monitoring. An adversary can use packet tracing to find the location of the source node, while the packets are being sent from source to sink for asset monitoring. Hence, endangering the asset. These issues are discussed in [1,2,3,4,6,7,8,18,19,20,21,22,23]. Figure 2 show categories that are associated with privacy issues in WSNs. Contextual privacy is the main issue of our interest and we presented a novel approach to protect.

### 1.3. Contribution

Although a lot of work has been done in the area of source location privacy, but there still is significant space for quality work to further enhance the SLP. We can summarize the novelties in our work, as follows.

We have proposed a scheme for source location privacy, for which we have assumed a WSN arranged in square grid having four quadrants with BS in the center of grid. We further assumed that all of the nodes know their relative location from BS in the WSN. When a node wants to report activity to the BS, it became source node. BS selects a fake source and a phantom source based on the location information of source node and the selection method is explained in Algorithm 1. Similarly, a circular zone, called the blast ring [20], around the BS contain nodes specialized for doing flooding inside the ring. The ring radius can be increased or decreased, depending on the situation. The nodes inside the blast ring are known as blast nodes. The terminology blast ring and flooding ring is interchangeably used throughout this text. The blast nodes on the edge of the ring, when it receives a packet to be delivered to BS, start flooding in a controlled manner. Flooding is done inside the ring within its diameter until the BS receives the packet. BS is single point where all of the sensing data accumulates and provides an easy point for back tracing the incoming packets path for source location identification. The concept of blast ring is used here to create hurdles for adversary by tracing back the packets from a single point (BS). Our proposed technique provides three levels of confusion to adversary, from tracing back the packets. First of all, a fake node is nominated, which sends packets toward BS. Secondly, phantom node is selected, which sends the packets to BS after it receive them from a random path from source node. The third level of the inclusion of blast ring that prevents back tracing from single point and adversary having to walk around the ring for tracing the path of incoming packets. Increasing the diameter of the blast ring increases the confusion for adversary, but the energy consumption also increases due to a widening of area containing flooding nodes. This is because, major source of energy consumptions are radio frequency transceivers [24,25,26]. All of these steps made it very hard for the outsider to find the source location.

The key contributions of our work can be summarized as:

Our proposed scheme has increased the safety period by 334% from the famous phantom routing and approx. 26% of the tree-based scheme [27]. The delivery ratio of the Dissemination in Wireless Sensor Networks (DeLP) is improved by approx. 26% from the tree-based scheme. DeLP utilizes 50% less energy utilization along with a reduction of delivery miss ratio of approx. 15 % as compared to the tree-based scheme. All of these factors show that our scheme performs better in all respects than the existing scheme.

The rest of the paper is organized, as follows: Section 2 describes different models. Section 3 discusses the related work. Section 4 describes our proposed work. The results are presented in Section 5. The analysis and evaluation are presented in Section 6, followed by conclusion and future work.

## 2. Models

### 2.1. Panda Hunter Game Model

**Figure 3:**
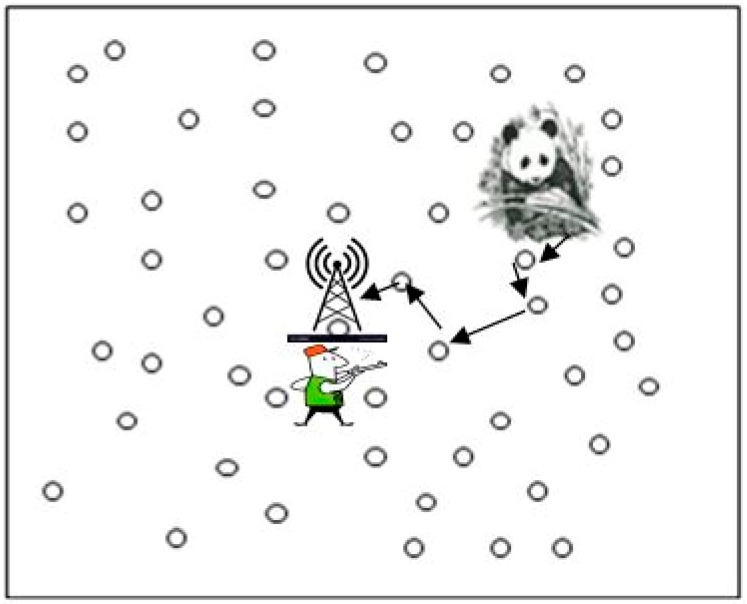
Panda Hunter Game Model.

In this paper, the famous panda hunter game model [3] is considered, as shown in Figure 3. This model is adopted for the WSN and deployed for the habitat monitoring of pandas. As soon as a node detects a panda, it became source node and keeps on periodically sending the observation via multi-hop routing techniques. The hunter uses the same network for finding the location of panda by using the necessary equipment for traffic analysis. The adversary captures a packet and backtrack the route to reach the originating node to capture the panda. It is assumed that the source node periodically sends the data with encrypted identity and the adversary cannot decrypt the message to find the information about source, but the message backtracking facility is used to reach the source node.

### 2.2. Adversary Model

The adversary is an illegal attacker that is equipped with sufficient energy resource along with memory and computational capabilities. The adversary is mobile and is well equipped with GPS, spectrum analyzer, and antenna. The adversary normally starts from the BS and, upon detecting a packet, calculates the angle or arrival to reach the immediate sender node. While waiting at a node, the attacker starts a timer and waits to receive a new packet. The attacker also records the current sensor node in the memory to overcome looping. The attacker moves to next node on receiving the packet before the expiry of the timer and calculating angle or arrival. In this way, he uses the backtracking technique to reach the source node. During back tracking at a certain node, if the attacker is not able to hear a new message and the timer is expired, the attacker needs to roll back to the previous node and delete the previous node from memory. The sensing radius of the attacker is assumed to be equal to the transmission radius of a sensor node. The attacker is supposed to be passive, which means that the attacker cannot provide any harm to the sensor nodes in the network. Algorithm 1 explains the strategy of the attacker. Table 1 summarizes the notations and symbols that are used throughout this paper.

**Algorithm 1** Adversary attack model1:  *Æ* starts as BS and start hearing for packets. When it overhears a packet from a node say node B;2: *Æ* move to immediate sender node and sets the timer ON for timeout interval $a^{\prime }$;3: *Æ* record new node in the memory to avoid looping (in this case new node is node B)4:  **While** (overhearing at ƥ*)*
**do**5:  **if**
*Æ* receives a packet from ŋ **AND** ѱ < $a^{\prime }$6: → ŋ 7: record ŋ in the memory 8: **break;**9: **else if**
*Æ does not* receives a packet from ŋ **AND** ѱ >=$a^{\prime }$11: delete ŋ from the memory (remove latest saved node) 12: **break;**13: **else**14: do nothing;15: **end if**16: **end while**

### 2.3. The Network Performance Model

The nodes in WSN are usually dependent on a battery source and the point of interest is reducing the network energy consumption to increase the network lifetime. A sensor node consumes energy when it is sending messages, receiving messages, idle listening, computing, or sensing the physical world. Out of these, sending and receiving the messages consumes most of the energy [24,25]. Apart from energy efficiency, other performance parameters for comparison may be safety period, throughput, and delivery miss ratio. Obviously, we cannot acquire excellent results of all of these performance parameters. One parameter efficiency may be increased on the cost of other. For example, if we want to increase the safety period, we need to design an excellent routing mechanism, which can result in reduced backtracking. This may also result in decreasing the energy efficiency as compared to shortest path routing. This means that we have to compromise few parameters to obtain good results for source location privacy.

Some major terminologies and parameters discussed in this paper are given below.

- The safety period is the time required by the attacker to backtrack and capture the panda, or in other words, the maximum time a panda stay at a source node before moving to another node.
- Throughput is the measure of total number of packets successfully received at the base station
- The network lifetime is shown in terms of rounds and it is the total life span of network from the establishment to the death of last node.
- Delivery miss ratio shows the measure of number of packets that are sent by the source node but not received by the base station.
- Total energy consumption is the measure energy consumed by sensor nodes during the lifetime of the network. It also includes energy consumption during transmissions, receptions, calculations, overhearing packets, and idle state.

## 3. Related Work

To address source anonymity in a network, a lot of work has been done in the past. Anonymous routing is a term that is used to hide the sender identity over the Internet. Better solutions for this are Chaum’s mixes [26] and onion routing [27]. In Chaum’s scheme, if a node wants to send data to another node, it is first sent encrypted to a central server, called the anonymizer, which then removes the source address from the packet and sends it to the destination. In this way the sender remains anonymous to the receiver.

In Onion, routing encryption on source routing is used, so that the source identifies the entire routing path from source to sink and the sent messages are encrypted in layers according to the intermediate nodes on the path. The next hop to send the message is identified by the present node by decrypting the message while using its own private key. This leads to hide the source identity because an intermediate node is aware of just immediate sender and receiver node and none of other node on the path.

Although the above-discussed two schemes are excellent, they cannot be implemented in wireless sensor networks, firstly because the layers of encryption are not feasible in memory, power, and processing limited sensor networks. Secondly, hop by hop transmission from source to sink in wireless senor network environment allows for an easy approach for adversary to perform traffic analysis and backtracking using spectrum analyzers and related devices. This may lead the adversary to the source node, because the encryption can secure the contents and cannot secure the contextual privacy of the network.

Back tracking if single path routing is used compromises source location privacy. Fake packet injection by the fake sources is proposed in [2]. The fake sources are required to generate the fake messages that are identical to the real messages and can also be encrypted to lead the adversary to fake source instead of real one. In [3], the idea of phantom routing is also proposed, in which a directed random walk phase is used to deliver the message to a phantom source and then single path or flooding routing may be used to deliver the message to the sink. Accordingly, packet tracking may lead the adversary to the phantoms source and the real source remains hidden. The limitations of phantom routing are identified in [18], where it may lead to the pre-termination of random walk phase due to the inappropriate selection of the random walk direction, and hence a dropdown in the performance may be observed in certain areas. The solution to the problem is proposed as to divide the neighbor set in four directions (E, W, N, S). The real source randomly selects one direction from the list for the random walk phase. However, by encoding the direction vector into the messages, the random walk is converted into self-adjusting random walk, even if the random walk is blocked in one direction. The self-adjusting random walk also allows for using a predetermined ratio to determine whether a random walk phase may be continued or aborted if the two directions are blocked. A random path might backtrack to itself after some time, so a loop may be formed. It is desired that the loops may be avoided and extend the path as long as possible to place the phantom source far away from the real source.

**Figure 4:**
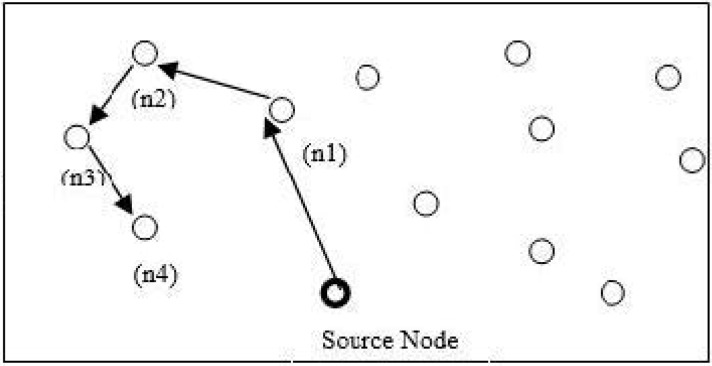
Showing a near to sink phantom node.

For this reason, Greedy Random Walk (GROW) is proposed for random walk phase in [4]. The idea of Bloom filter is used to store all of the current neighbors in the forwarding packet. When the next hop randomly picks up one neighbor, it checks whether the neighbor entry is present in the filter or not. To decrease the chance of backtracking, each sensor keeps a Bloom filter to store those neighbors that have already participated in the forwarding. Each time a sensor forwarding a packet stores the immediate upstream and downstream nodes. The author admits that the message delivery ratio is longer enough and the proposed design is somewhat good for small networks, but not realistic for large networks. Apart from these two, we have also identified other limitations. First, as the proposed design allows for the adversary to recover significant routing information if the message is captured, the source location may be compromised. Secondly, the author does not address a situation if a message reached a node that has neighbors who have already in visited list. For example, Figure 4 clearly shows this situation to understand that a message from source node reached ultimately to node *n4,* but the loop can only be avoided if the random walk phase is terminated at *n4* and the shortest path routing phase starts, but this will bring the phantom source (*n4* in this case) near to the real source. This seems to be a limitation of this scheme.

**Figure 5:**
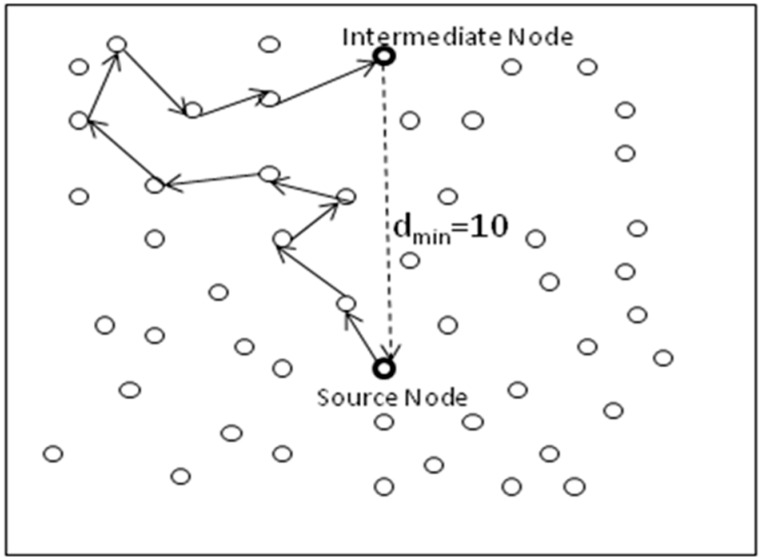
Showing d_min_ from source to intermediate node.

Source location privacy through Routing to a Random Intermediate Node (RRIN) and source location privacy through Angle-Based Multi-Intermediate nodes is proposed in [19]. The sensor nodes are assumed to have knowledge regarding their relative locations and their adjacent neighboring nodes. In RRIN, the intermediate node is randomly selected by the source node and should be at least d_min_ distance away from the source node to make it difficult for the adversary to obtain real information regarding source node, as shown in Figure 5. The intermediate node is the last node of random walk phase after which shortest path routing may be adopted towards sink. The intermediate node is selected on the basis of relative location of the source node, so as to make it possible to select the intermediate node as far as possible.

In source location privacy through Angle-Based Multi–Intermediate Nodes, the source node preselects the intermediate nodes before sending the message and information about the intermediate nodes is stored in the message header before forwarding by the source node. This places a high risk on the security side, as the adversary may find all of the routing information from a message by staying close to the SINK. To handle this issue, it is the previous information regarding the intermediate node that will be deleted from message header before forwarding a packet by an intermediate node. Source location privacy through Angle Based and Quadrant based Multi-Intermediate Nodes have been discussed by the authors and the results show that the adversary could find the last intermediate node, but cannot find the source node. Since each sensor node has knowledge of its adjacent nodes and may not have accurate information about an intermediate node multiple hops away from the source. It may not even exist, so it is assumed that the last node in the routing path adjacent to the intermediate node will become the intermediate node.

A type of traffic analysis attack in random walk phase is proposed in [28] to find the source location in a planner shape sensor network with sensors that are uniformly distributed inside the plane. The monitoring stations (adversary) are assumed to be at the outer boundary of the network. The network has restricted access to anyone, except the network owner. The monitoring stations gathered the information of the packets issued by the source that hit the boundary for the first time, called the exit distribution. An algorithm is used to find the source location using the exit distribution. The problem that is found in the proposed scheme is that the author totally ignored the idea of fake sources in the sensor network. The attack can be easily launched by the injection of fake source, having a high probability of sending messages to the boundary nodes, which may lead the attacker to the fake sources instead of the real one.

In [6], a realistic model of attacker is considered in a habitat monitoring application of pandas. The attacker has installed many observation monitoring devices. A hotspot region is a region where more pandas are found. It is clear that more packets will be generated in the hotspot regions. The attacker can exploit the hotspot locating attack. The author proposed the cloud-based scheme to counter the hot-spot locating attack. In the cloud is composed of an irregular pattern of traffic with fake traffic. The idea is to confuse the attacker by mixing the real traffic with fake traffic, so that the real source can remain hidden in the fake traffic or cloud. These clouds can be further mixed to increase the confusion for an attacker. The creation of this much fake traffic can increase energy consumption many folds. Accordingly, the clouds may only be formed when real data transmission is needed and then deactivate it accordingly.

In [7], a tree-based routing scheme is proposed for achieving source location privacy. This scheme produces diversionary routes for misleading the attacker. There is a fake source on each end of the diversionary route. The idea was to improve the energy efficiency by utilizing the energy in non-hotspot regions. In this scheme, many paths are created and the packets paths cross themselves, which creates problems for attacker. The inclusion of many paths and crossover increases the path length for packets, which introduces packet delays and reduces the packet delivery ratio. Many diversionary routes involve more nodes in transmission and reception process, so the overall energy consumption of the network increases many folds when compared to phantom protocol.

The random packet forwarding technique is used in [22] for SLP. For the network model, it uses the two-dimensional coordinate system. The network consists of four quadrants with the sink placed in the first quadrant. The sink first broadcasts its location to all nodes. Each node creates two sets of reference coordinates using the sink and nodes own location. Df is a constant directivity factor that is present at each node. When a node receives a packet, it generates a random number and compares that number with the Df. The random number is then compared with the Df and one of the reference coordinates is selected on the basis of greater or less than the Df. After this, the shortest path is calculated using routing table and the packet is forwarded to the selected reference coordinate. This technique utilizes more memory on nodes due to the use of routing tables. Further packet latency is involved due to the extra processing involved due to routing tables. Energy consumption is also increased due to the extra hops between source and sink.

From the existing work, we know that, for protecting SLP, we need to add some confusion for the attacker. This may complicate the routing process of the packets from source to sink. Other nodes, like fake, phantom, receptor, and intermediate, to name a few, are considered in the literature. To increase SLP, the tradeoff of increasing energy consumption also exists. Out of many of the proposed solutions for energy efficient WSN, few are energy efficient routing, network balancing, considering residual energy of nodes, and energy efficient cluster head selection. References [29,30,31,32,33] discusses some techniques for energy efficiency in WSN.

## 4. The Proposed Work

**Figure 6:**
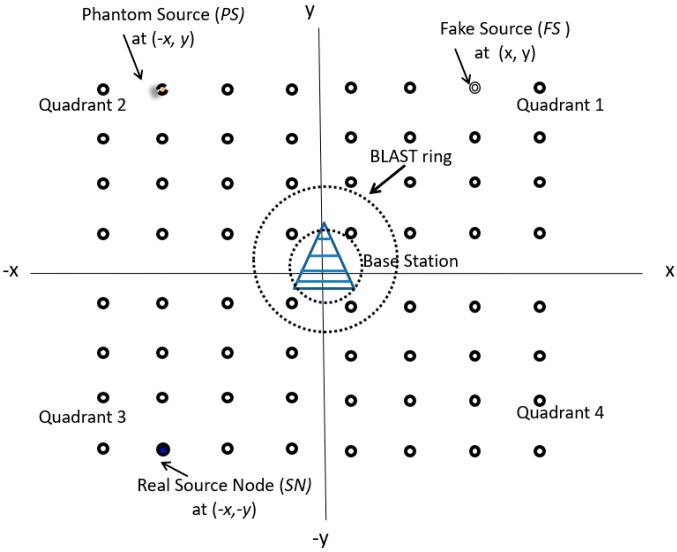
Schematic of proposed model.

In this work, we have proposed an enhanced source location-based privacy technique for wireless sensor network. At the time of network launching, all of the nodes are informed regarding their relative location from the base station using the network broadcasting [25]. It is assumed that all of the sensor nodes are evenly distributed in the surveillance area, as shown in Figure 6.

**Figure 7:**
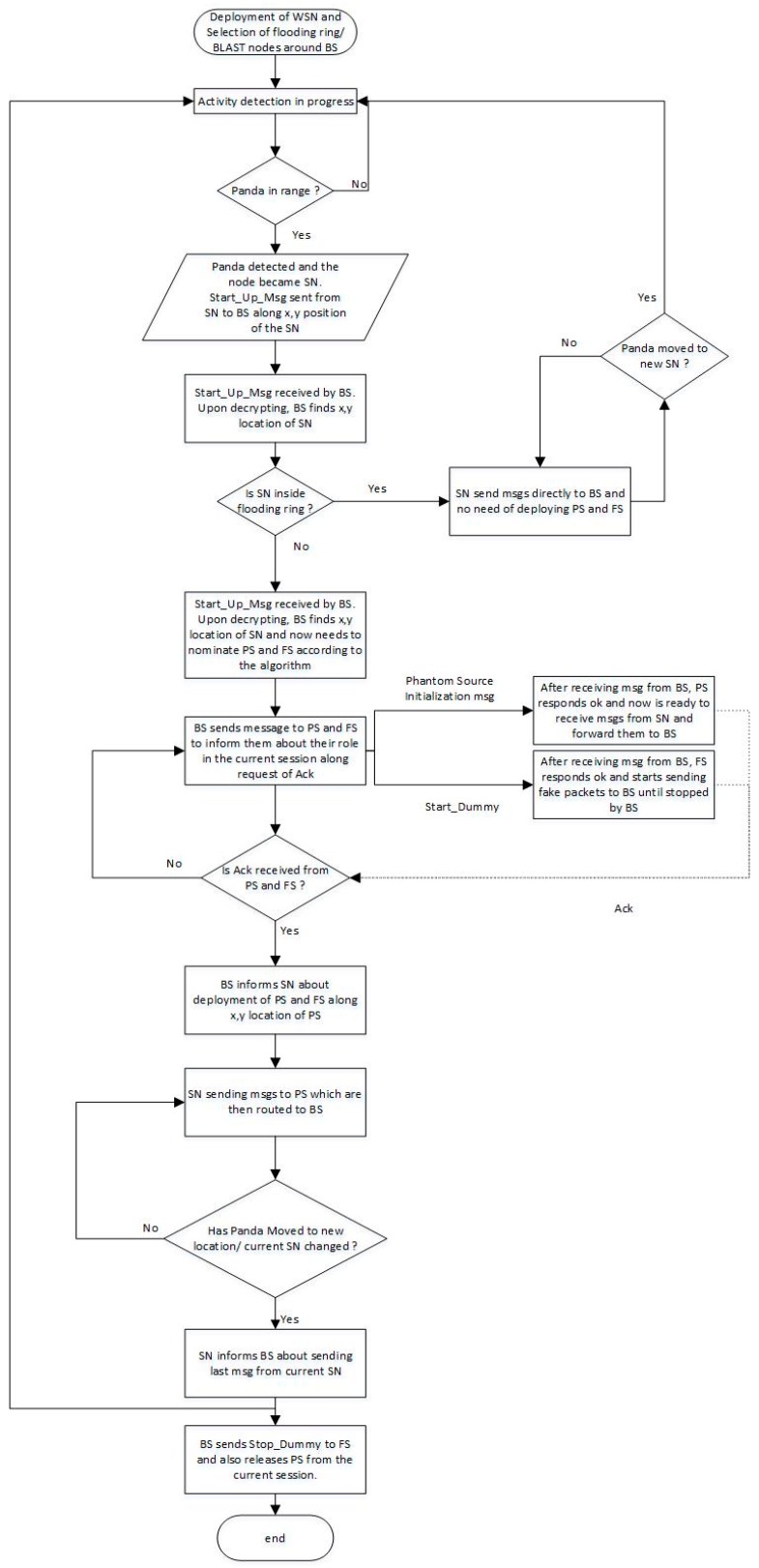
Flow chart of our proposed model.

We have assumed a square grid having four quadrants and a uniform distribution of sensor nodes with BS at the center of the grid in our proposed technique, as shown in Figure 6. Our algorithm has four main steps: (i) Network deployment and selection of BLAST ring by BS around itself; (ii) Activity detection and identification of Source Node (SN); (iii) Selection of Fake Source (FS) by the BS from the information of SN; and, (iv) Selection of Phantom source (PS) by the BS from the location information of SN. The flow chart is presented in the Figure 7.

The nodes near the BS are named flooding/BLAST nodes and forward messages to the BS using flooding. In this way, the BS from many nodes may receive a single message. This can help to increase the privacy, because the attacker may not be able to back track from the single aggregation point. The attacker needs to go at the edge of the blast zone/ring for backtracking the packets. For this, he needs to go around the blast ring for catching a packet hence increases his efforts and time utilization in attacking. By increasing the diameter of the blast region, the safety period will increase, but the price to pay is more energy consumption due to more nodes being involved in flooding. Similarly, we can take example of increasing the diameter of the flooding ring, so that it encompasses the whole network. Upon activity detection, the packets are flooded in the whole network and for attacker the packets will be coming from every side. This will restrict the attacker to take correct decision in finding the source node and location privacy will be ensured. Accordingly, there is a tradeoff between increasing the privacy and energy consumption.

### 4.1. Network Model

Our network model consists of 400 nodes that are uniformly distributed in the sensing area of about 400 × 400 square meters. The whole sensing area is divided into four quadrants with the base station in the middle. Each quadrant is assumed to have an equal number of nodes. The nodes are assumed to be static and each node knows its relative position (x, y) from the base station. The nodes are programmed to send messages to the base station on a hop by hop basis. This means that, once a node detects an event, it starts reporting by using other nodes in the network. All of the nodes have equal chances to become a source node, but for the sake of clarity we are assuming only one source node at a time in our model.

In our network, when a node detects an activity, that node became source node. There is no need of deploying the phantom source and fake source if the source node is inside the flooding ring. The source node report to the BS using the flooding technique. If the source node is outside the flooding ring, then again source node does not start reporting directly, but it has to first choose a fake source and a phantom source to confuse the adversary, and it is discussed in Algorithm 1. The fake source is selected from the opposite quadrant. Opposite here means that 1 and 3 are opposite and similarly 2 and 4 are opposite. The fake source is selected from the quadrant on the left or right side of the source node, which depends on the x, y position of the source node and it is selected on the basis to increase the distance between source node and fake source.

### 4.2. Importance of Flooding Region in our Technique

The idea of deploying flooding region is very helpful in increasing source location privacy because of two reasons.

- Firstly, most of the techniques that are present in the literature provide poor source location privacy if the source node is present near the BS, because backtracking is easy. However, in our technique, if the SN is inside the flooding ring, then, due to the flooding technique, if an attacker is near the BS and wants to backtrack a packet, then it will be almost impossible to reach the actual destination. This is because the packets are coming from all of the neighboring nodes and it makes backtracking a tough job.
- Secondly, as BS is the region of convergence for all packets, the backtracking is normally done from BS. However, as BS is surrounded by flooding nodes, it is not possible for the attacker to start the backtracking from BS, therefore the attacker needs to come out of the flooding area to continue backtracking. This again safeguards the source node present inside the BLAST region. If the source node is outside the flooding region, the attacker still needs to search for incoming packets towards the BS (flooding region) by moving around the boundary of whole flooding region. This will waste his energy and time, hence providing more safe time for the asset to move to new location.

### 4.3. Algorithm for DeLP

**Algorithm 2, for selection of fake and phantom source for different scenarios of real source places other than inside blast ring**1.    if source node is in quadrant 1:**Input:** source node quadrant x and y position P_SN_ = (x,y)**Output:** Fake and Phantom source quadrant along (x,y) position*FS*
*→ Q_3_ with P_FS_ = (−|x|,−|y|)****If***
*|x| > |y| then**PS*
*→ Q_2_ with P_PS_(−x,y) = P_SN_(|y|,|x|)****Else***
*PS*
*→ Q_4_ with P_PS_(x,−y) = P_SN_(|y|,|x|)*2.    if source node is in quadrant 2:**Input:** source node quadrant x and y position**Output:** Fake and Phantom source quadrant along (x,y) position*FS*
*→ Q_4_ with P_FS_ = (+|x|,−|y|)****If***
*|x| > |y| then**PS*
*→ Q_1_ with P_PS_(x,y) = P_SN_(|y|,|x|)****Else***
*PS*
*→ Q_3_ with P_PS_(−x,−y) = P_SN_(|y|,|x|)*3.    if source node is in quadrant 3:**Input:** source node quadrant x and y position**Output:** Fake and Phantom source quadrant along (x,y) position*FS*
*→ Q_1_ with P_FS_ = (+|x|,+|y|)****If***
*|x| > |y| then**PS*
*→ Q_4_ with P_PS_(x,−y) = P_SN_(|y|,|x|)****Else***
*PS*
*→ Q_2_ with P_PS_(−x,y) = P_SN_(|y|, |x|)*4.    if source node is in quadrant 4:**Input:** source node quadrant x and y position**Output:** Fake and Phantom source quadrant along (x,y) position*FS*
*→ Q_2_ with P_FS_ = (−|x|,+|y|)****If***
*|x| > |y| then**PS*
*→ Q_3_ with P_PS_(−x,−y) = P_SN_(|y|,|x|)****Else***
*PS*
*→ Q_1_ with P_PS_(x,y) = P_SN_(|y|,|x|)*

### 4.4. Packet Routing

#### 4.4.1. Packet Routing for the Case of Source Node Inside Flooding Ring

There is no need of selecting fake node and phantom node if the source node is selected inside the blast ring (flooding region). The packets are delivered using simple flooding technique. The source node sends the packet to its neighbors and neighboring nodes intern broadcast to others. If a node receives a packet which was already transmitted by that node, it will be dropped immediately.

#### 4.4.2. Packet Routing for the Case of Source Node Inside Flooding Ring

If the source node is identified in a region other than flooding ring then the proposed Algorithm 1 is followed and the selection of FS and PS is mandatory. If panda is detected in a quadrant (e.g., quadrant 1), a single message is generated by the source node and sent to the BS. A single message is impossible to back track, so there is no need to worry about source location detection. The node that detects reporting activity becomes SN. FS and PS are not selected purely by the random method because there is a chance that all the three sources or any of the two may be selected in the same quadrant. This will lead to produce more traffic in one quadrant and the attacker may find it helpful in searching for SN. Therefore, the selection process involves the algorithm with the aim of producing enough distance between the sources.

**Figure 8:**
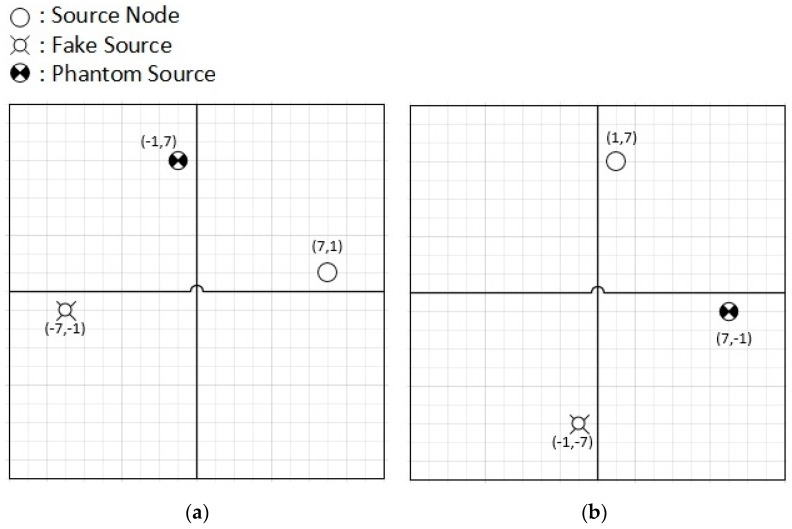
(**a**,**b**) are showing two different situations of Source Node (SN) in first quadrant.

Upon the detection of panda in the first quadrant, the fake source is deputed in opposite quadrant (third quadrant in this case). The Phantom node is selected in either the second or fourth quadrant, and it depends on the distance of the source node from the lines separating the quadrants from each other. The reason behind this is to increase the distance between the source node and phantom node. This is shown in the following Figure 8 for more clarity.

The signs used in the Figure 8 represents:

Upon the detection of panda in the first quadrant with x, y location (7, 1), as shown in Figure 8a, the fake source (FS) is deputed in opposite quadrant (third quadrant with x, y location (−7, −1), only the signs changed due to change of quadrant) in this case. The Phantom source (PS) is selected in either the second or fourth quadrant, and it depends on the distance of the source node from the lines separating the quadrants from each other, which also produces some level of randomness in the selection of PS. The quadrant selection of PS is not strict, but it depends on the location of the SN, so that the aim of increase of distance between the source node and phantom node is achieved. If source node is near to horizontal line (|x| > |y|), as shown in Figure 8a, the SN is near to quadrant 4 with respect to quadrant 2. Accordingly, the PS is selected in quadrant 2. If the x, y location in the second quadrant if taken as same as SN, which comes to be (−7, 1), the PS becomes near to FS, which may not be a good decision. For this purpose, to keep the location of PS away from FS, the x and y locations are interchanged, now the location of PS will be (−1, 7). This leads to keep the three sources away from each other to create more hard work for attacker.

For the case of Figure 8b, the source node is in quadrant 1 with (x, y) values of (1, 7). The location of the fake source is straight forward third quadrant, with same x, y values and signs of the quadrant selected, that is x and y both being negative, so the location of fake source is (−1, −7). For phantom node selection, the x, y values of source node are taken into consideration. As the (x, y) values of source nodes are (1, 7), the location of the phantom node will be (7, −1). The –ve sign with y, because it is fourth quadrant, where y is –ve.

In this way, when an activity is detected, the activity detecting node becomes SN and FN is deployed in the opposite quadrant. The PS deployment is done according to the algorithm, so that all three sources are away from each other.

### 4.5. Energy Consuption Model

Table 2 presents the network parameters.

We considered the typical energy consumption model in [5]. Here, the *E_t_* is the transmission energy consumption and is described in Equation (2), while *E_r_* is for receiving energy consumption and it follows Equation (3).

(1) $$E_{t}=lE_{elec}+l\varepsilon_{fs}d^{2},ifd<d_{o}$$

(2) $$E_{t}=lE_{elec}+l\varepsilon_{amp}d^{4},ifd>d_{o}$$

(3) $$E_{r}=lE_{elec}$$

### 4.6. Timing Diagram

The timing diagram is explained in this section in order to understand it. When SN has to report an activity, it sends a single message, called *Start_Up_Msg*, to the BS. A single packet is almost impossible to back track and it provides a safe way to inform the BS about the activity initiation. This packet has information regarding the source ID, its position (x,y), and session initiation information, along with an acknowledgement request to start the session. The blast nodes receive the *Start_Up_Msg* and it is delivered to BS using flooding. The *Start_Up_Msg* is similar to ordinary messages, but, upon decryption, the BS knows that this message means that a node has detected some activity and wants to report and this reporting may be continued for some time (like the pandas may stay longer at the source node, so the source node has to report regularly). This continued reporting cannot be done on direct route, as the adversary can easily back track the packets and the chances of locating the source node increases.

The BS, upon receiving *Start_Up_Msg*, selects a fake source (FS) in opposite quadrant according to the algorithm. BS deputes the FS by sending a message, called Start_Dummy, along a request of acknowledgement. The FS has to send dummy messages to the BS until it receives Stop_Dummy message from BS. FS has to send the messages on a natural rate as set on other nodes are using and they are delivered to BS using random walk. The messages from the FS helps to misguide the attacker. Meanwhile, the BS also needs to select the PS according to algorithm. BS sends phantom node initialization message to the PS along a request of acknowledgement. BS informs the PS about its role that it needs to receive the packets from SN and then send it to the BS while using random walk. The BS, upon receiving acknowledgement from FS and PS, inform SN to start reporting. The reporting is done via the phantom source. After the SN stops reporting, the BS informs the selected FS to also stop sending dummy messages (Stop_Dummy) and it also informs the PS about its release from current communication session.

### 4.7. DeLP for Attacker

Let us now explain the whole procedure of attacker interested in capturing the source node (panda). It is clear in Algorithm 2 that, as all network traffic converges to the BS, the adversary takes a start by backtracking packets from the BS. If there is no activity, there are no messages and adversary has to wait. As the sensing activity starts the packets from the FS and PS creates flooding in the blast ring. The adversary upon backtracking realizes that the packets are coming from all nodes. This puts the first hurdle for him. The only solution for him is to find out the outer boundary of the flooded area and wait there for incoming packets. Suppose that he found the outer boundary and reached there after some time, then another problem that he faces is that he has to start from one point and move around the outer boundary for any packet detection. Increasing the radius of flooding ring increases problems for adversary.

Now, we consider that adversary reaches the ring boundary in quadrant 1, as shown in Figure 6. His spectrum analyzer starts detecting packets. Upon back tracking, the adversary ends up with the fake source. After this search, he has to returned to ring boundary and start another search.

Suppose that travelling on the ring edge, the adversary reaches quadrant 2 and detected some packets coming towards BS. The adversary again starts searching but unluckily ends up with phantom source.

## 5. Results

**Figure 9:**
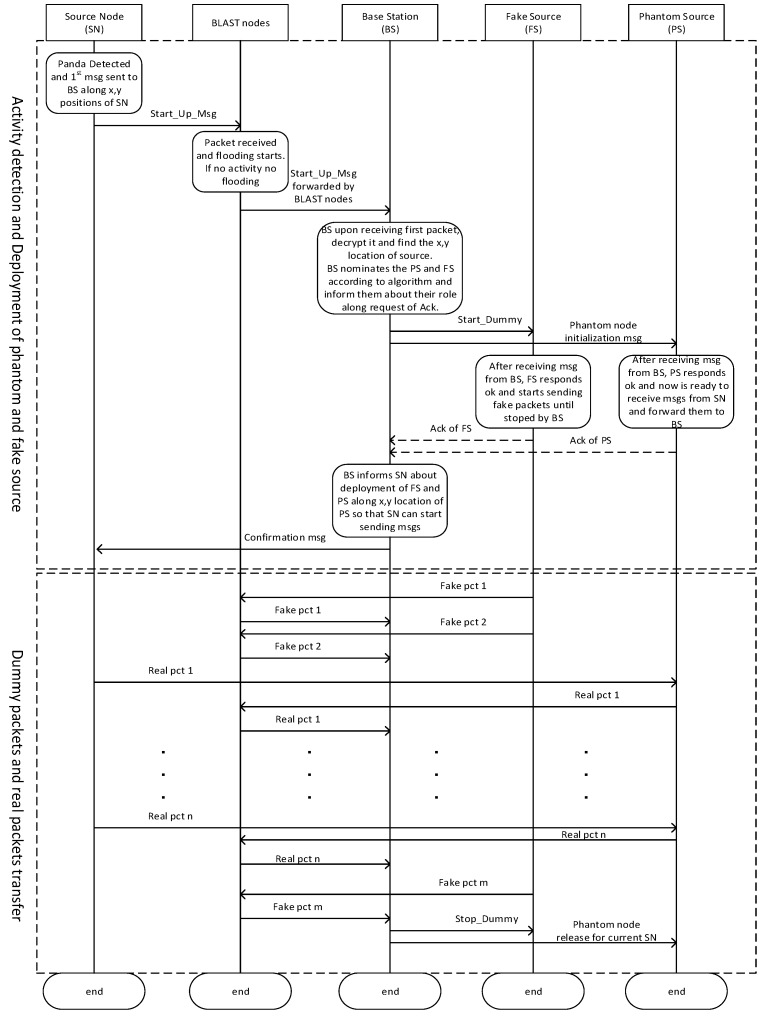
Timing Diagram of the proposed technique from the deployment till release of the SN.

**Figure 10:**
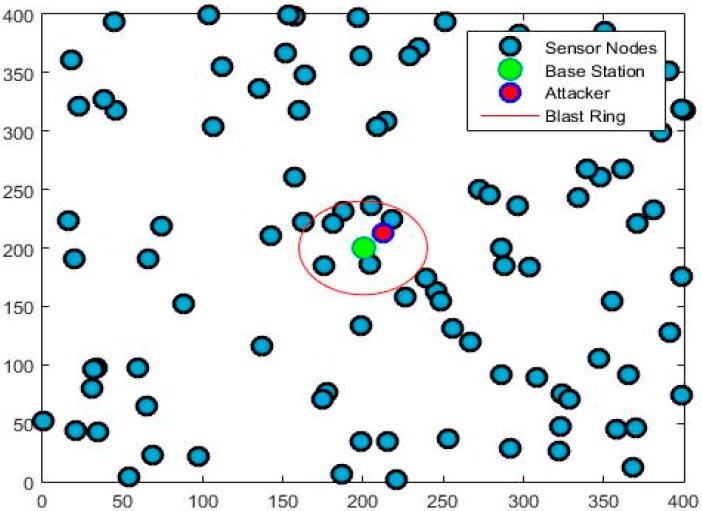
Our proposed simulation model.

In this section, we evaluate the performance of our proposed technique in MATLAB as depicted in Figure 9. Our model consists of 400 × 400 m square with the base station in the middle. One hundred nodes are randomly deployed in the area. The blast ring is set with a radius of 40 m around the base station, as shown in Figure 10. Table 2 discusses the network parameters.

The phantom routing is considered as a baseline in our simulations. We have seen that the phantom routing is mostly used in the literature for comparisons. Phantom routing provides good energy efficiency and delivery ratio, but is not efficient in providing excellent source location privacy. In order to achieve a high level of SLP, it is evident from the literature that we have to compromise energy consumption. This tradeoff is acceptable if we have such WSN applications, where the location privacy is of highest importance.

We have three main sources in our proposed model: the source node, fake source, and phantom source. The data from all of the sources are aggregated towards the base station. The attacker normally resides near the BS at the start and then use back tracking in order to find out the source node. Algorithm 1 discusses the backtracking procedure that was used by the attacker.

### 5.1. Throughput

**Figure 11:**
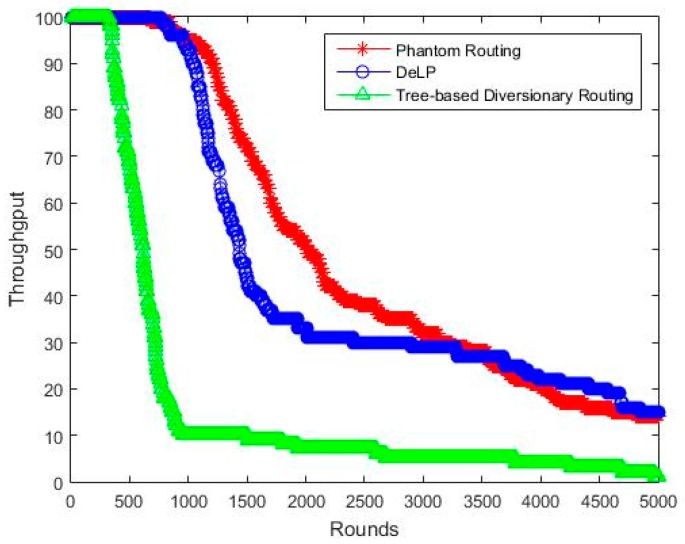
Delivery ratio Vs Network lifetime (rounds).

Figure 11 compares the delivery ratio of our proposed scheme with phantom routing [3] and tree-based diversionary routing [7]. The results show an early decay of packet delivery ratio in tree-based, followed by DeLP and phantom routing. As phantom routing is simple, with no extra paths, so the delivery ratio performance is good when compared to the other two schemes, but in return it compromises the safety period. The delivery ratio of the tree-based scheme is worse because of long routing paths and due to large number of diversionary routes having fake nodes, which results in the collision of packets. The extra-long paths also introduce delivery latency.

Table 3 shows the comparative analysis of packet delivery ratio. The results show that the delivery ratio of DeLP has an improvement of more than 600% as compared to the tree-based scheme. The main reason behind the good delivery ratio of DeLP is that the source node, phantom, and fake node exist in different quadrants. This reduces the chance of overlapping paths, and hence reducing the chances of packet collisions. On the other hand, phantom routing shows an improvement of only about 10% in terms of packet delivery ratio when compared to DeLP. However, this comes with paying a big cost of compromising source location privacy, where DeLP shows an improvement of more than 300% than phantom scheme. Hence, for an obtaining such a big increase in SLP of more than 300%, compromising a 10% in packet delivery ratio is not a big deal. The results indicate that the DeLP outperforms the tree-based scheme in throughput while providing efficient source location privacy.

### 5.2. Safety Period

**Figure 12:**
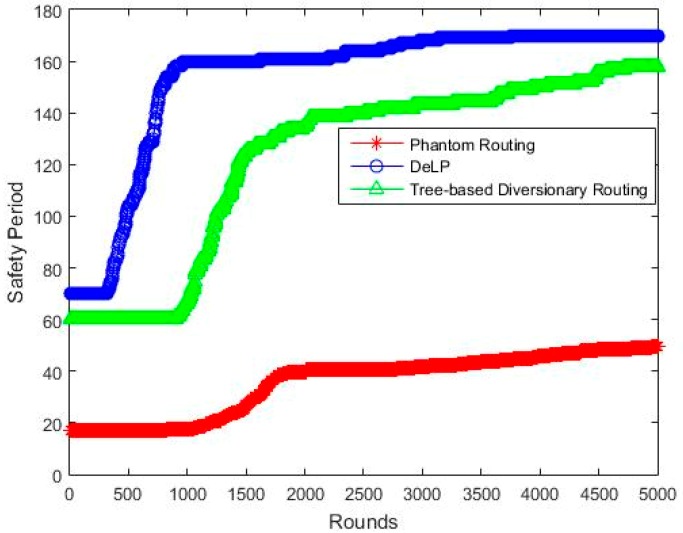
Safety period time Vs Network lifetime (rounds).

Figure 12 shows the safety period of the analyzed schemes. The results show a lesser safety period than the tree-based scheme, as the phantom scheme cannot not create enough confusion for adversary. The tree-based scheme produces many paths and each path ends with a fake source. The safety period of tree-based scheme is mostly dependent on the number of diversionary paths. As the number of diversionary paths increases, so does the safety period. However, increasing the number of paths comes with a great cost of increasing the overall energy consumption of the network.

Table 4 shows the comparative analysis of safety period. Out of the three schemes, our proposed scheme shows excellent results outperforming the other two schemes. The results indicate that DeLP shows a tremendous increase of safety period approx. 334% more than the phantom routing. It is also evident that DeLP performs better than the tree-based scheme, by showing 26% more safety period. This is because the fake packets and real packets are coming from different sides, while the real source node resides at a different quadrant. In addition, the BS is surrounded by a flooding nodes ring, which further confuses the attacker. As the attacker near the BS is receiving packets from all sides, he is considerably confused about which packet to backtrack. Even if he decides to backtrack anyone, he may end up with either fake or phantom source, which increases the safety period. The performance of tree-based scheme is also good when compared to the phantom scheme, but is still less than our proposed scheme.

As our technique constitutes of three layers of confusion, namely the blast ring, phantom source, and fake source. The major contribution towards the saftey period is offered by the blast ring, followed by phantom source, and then fake source. Different experiments revealed that 60% of the saftey period contribution was made by the blast ring alone. In the remianing 40%, the phantom source contributes 24% alone, while 16% was contributed by the fake souce.

### 5.3. Energy Consumption

**Figure 13:**
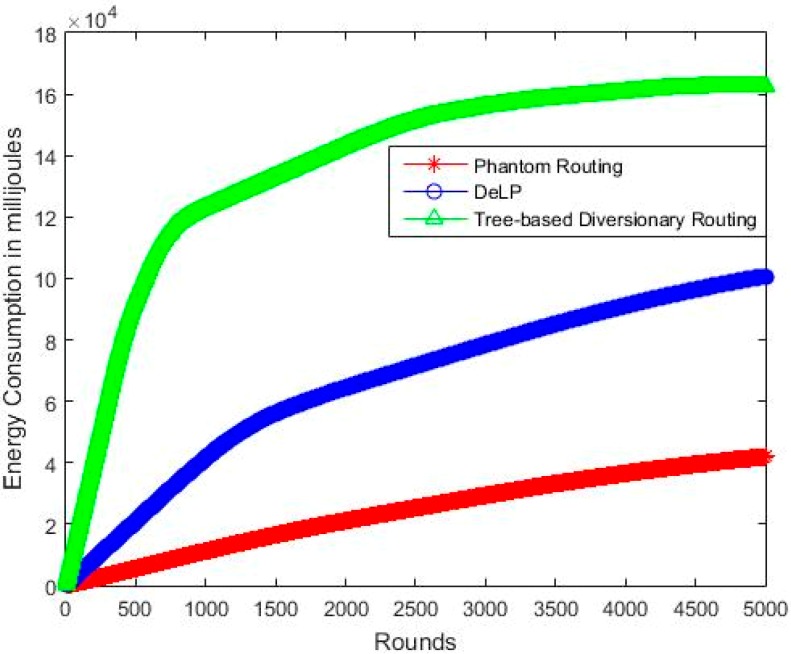
Net Energy consumption Vs Network lifetime (rounds).

Figure 13 shows the energy consumption of the analyzed schemes. Phantom routing shows less energy consumption than the other two, but it results in a lesser source location privacy. The energy consumption is dependent on the path length between source and sink. Optimal results for energy consumption can be achieved using shortest path routing, but the SLP will be compromised. We need to compromise energy consumption in order to have a good safety period. This tradeoff is acceptable, because we need to improve the safety period and need to increase confusion for the attacker. The increase of energy consumption is worthy in the case of such applications where we need to monitor valuable assets and safety is of highest importance. In comparison to phantom routing, our scheme guarantees that the PS will not be deputed in the same quadrant of SN, but in the adjacent quadrant with the aim to make them far apart to increase the path length. This is one of the factors in increasing SLP and one of the reasons of more energy consumption than simple phantom routing.

Table 5 shows the comparative analysis of the schemes for energy consumption. As we have considered phantom routing as a baseline scheme, therefore the other two schemes have higher energy consumption than this. However, in return, they both provide enhanced SLP than the phantom scheme. The results show that the energy consumption of DeLP is approx. 50% of that of tree-based routing. This concludes that DeLP provides about 26% improvement in safety period by consuming 50% less energy than tree-based routing. This makes our scheme efficient in terms of safety period and energy efficiency. Tree- based scheme uses many diversionary paths, which increase the overall path length of the packets, hence resulting in an increase of energy consumption. DeLP also uses long paths and a flooding ring along fake source, which increases the energy consumption. Tree-based and DeLP shows an increase of about 4.4 time and 1.7 time more energy consumption than the phantom routing.

### 5.4. Delivery Miss Ratio

**Figure 14:**
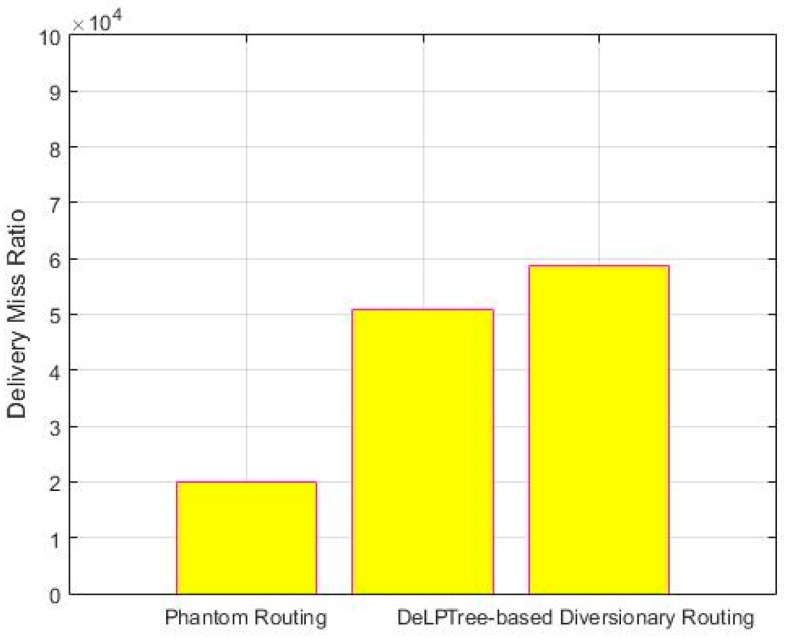
Comparison of Average delivery Miss ratio.

It shows the ratio of number of packets that are sent but not received at BS. Figure 14 shows the delivery miss ratio of the three schemes. Less confusion in the network results in less packets loss. The Tree-based scheme has highest packet miss ratio due to many diversionary paths. These paths cross each other, which results in packet collision, hence increasing the miss ratio. DeLP also employs a flooding region, which plays a role in packet collision.

The comparative analysis that is shown in Table 5 shows that, although DeLP provides a greater safety period of about 26%, along with a reduction of energy consumption to 50%, still it offers 15% lesser delivery miss ratio as compared to tree-based scheme. Due to the reason discussed above, the delivery-miss ratio of tree-based scheme and DeLP is approx. 1.9 times and 1.5 times, respectively, as compared to phantom routing.

## 6. Analysis and Evaluation

Our proposed technique provides three levels of security to the source nodes. First of all, the advantage of blast ring is that it confuses the attacker in a flooded environment. Secondly, if the attacker somehow managed to come out of the ring (ring edge), then he has to utilize his energy on tracking a big ring for incoming packets instead of tracking back from a single BS. Back tracking towards the FS places more hurdles for attacker. Similarly, the last chance of tracking packets coming towards blast ring is from phantom source and upon tracking that path the attacker still cannot locate the SN. This provides a big safety period for the source and, in the case of a mobile target, there is a big chance that the location of the target has already changed.

Our proposed approach has improved the source location privacy with a very little increase in the network traffic or energy consumption as compared to other techniques especially proposed in [5,6,7]. We are neither creating multiple fogs nor creating multiple diversionary paths for the adversary’s confusion, which actually increases the traffic and energy consumption. In our scheme, we have deputed some specialized nodes inside the blast ring for flooding, but they are not significantly contributing towards the increase in the network traffic and energy consumption.

The overall comparative analysis shows that DeLP proves itself to be an effective scheme in the considered performance metrices. DeLP outperforms tree-based scheme in all four performance metrices. It provides 26% more safety period, 600 % more throughput, 15% less packet delay and 50% less energy consumption when compared to the tree-based scheme.

In our area of research, we are more conscious about securing the location of source node, as it can compromise can result in a loss of valuable assets for who is monitoring the network is deployed. Routing techniques with the shortest path or techniques that cannot make confusion for an attacker cannot perform well for SLP. Techniques, like phantom routing, are one of the earliest techniques used for SLP uses simple architecture. This technique performs well as far as energy consumption, packet delay, and delivery ratio are concerned, but does not provide enough location privacy. The comparative analysis shows that DeLP provides 334%, while tree-based scheme provides about 242 % more safety period than phantom routing.

## 7. Conclusions and Future Work

In this paper, we presented an enhanced scheme for source location privacy, which provides three levels of security. After network deployment, the BS selects a ring of flooding nodes around itself. This flooding ring helps in confusing the eavesdropper, as the packets are converging towards the BS from all sides due to flooding. This reduces the capability of attacker to directly backtrack the packets from the BS and he needs to go around the whole flooding ring for backtracking. Similarly, the deployment of fake source is in an opposite quadrant of SN and the phantom source is in one of the adjacent quadrants. The idea was to increase the distance between the fake and phantom sources with respect to SN. This guarantees that the traffic is coming from different parts of the network towards the BS to put hurdles for the attacker in backtracking the SN. Quadrants also help in increasing the safety period. The results show that our proposed scheme has 50% less energy consumption, 26% more safety period, 15% less packet delivery delay, and six times more packet delivery ratio as compared to the tree-based scheme. We have also used the phantom scheme for comparison and our scheme provides 334% more safety period than it. However, it provides less energy consumption and packet delivery delay, and has more packet delivery ratio than our scheme due to the simplicity of architecture and reduced traffic.

Energy harvesting WSNs is a hot area and being investigated by many researchers to increase the lifetime of the networks. The main power source of wireless sensor nodes is their battery, so compelling it to the extra-required communication sessions may quickly drain it. This may cause the nodes isolation from the network due to the exhaust of its power source and may also result in the network. However, it is possible to utilize the solar cells with nodes in the blast ring of our wireless sensor network to meet the power demands and extend the life of the network [23]. This may cause a bit of an increase in the size of the wireless nodes due to the implantation of the solar cell. A credit card size solar cell may help in improving the power issue in areas having more sunny times (small winter seasons), but the size of the cell will mainly depend on the seasonal exposure of the nodes to sun. This feature may be very suited for the nodes inside the blast ring as an extra power source. The installation of credit card size solar cells on wireless nodes (especially nodes inside blast ring) is our future work to further enhance the network lifetime.

## Figures and Tables

**Table 1:** Important notations used in this work.

Notation	Meaning
*A, (x,y)*	Geographic area, location inside A
*SN*	Real source node
*FS*	Fake source node
*PS*	Phantom node
*BS*	Base Station
*SN*	Real source node
*Æ*	Adversary
*ƥ*	Present node
ŋ	New node
ϥ	Previous node
→	Move to
ψ	Wait time
*P_SN_*, *P_PS_*, *P_FS_*	Position of Source Node, Position of Phantom Source, Position of Fake Source respectively
*Q_1_, Q_2_*, *Q_3_*, *Q_4_*	Quadrant 1, Quadrant 2, Quadrant 3, Quadrant 4 respectively

**Table 2:** Network Parameters.

Parameter	Value
Threshold distance (*d_o_*) (m)	87
*E_elec_* (nJ/bit)	50
*E_fs_* (pJ/bit/m^2^)	10
*E_amp_* (pJ/bit/m^4^)	0.0013
Initial energy (J)	0.9

**Table 3:** Comparison of packet delivery ratio after equal intervals.

S.No	Name of Protocol	At 1000 Rounds	At 2000 Rounds	At 3000 Rounds	At 4000 Rounds	At 5000 Rounds	Average
1	Phantom	0.95	0.51	0.32	0.2	0.15	0.426
2	Tree-based Diversionary Routing	0.1	0.07	0.05	0.04	0.01	0.054
3	DeLP	0.93	0.33	0.29	0.22	0.15	0.384

**Table 4:** Comparison of safety period after equal intervals.

S.No	Name of Protocol	At 1000 Rounds	At 2000 Rounds	At 3000 Rounds	At 4000 Rounds	At 5000 Rounds	Average
1	Phantom	17.35	37.65	41.85	45	47.45	37.86
2	Tree-based Diversionary Routing	66.9	134.8	142.8	149.7	153.5	129.54
3	DeLP	160	162	166	167	168	164.6

**Table 5:** Comparison of energy consumption after equal intervals.

S.No	Name of Protocol	At 1000 Rounds	At 2000 Rounds	At 3000 Rounds	At 4000 Rounds	At 5000 Rounds	Average
1	Phantom	1.124	2.12	2.96	3.6	4.16	2.7928
2	Tree-based Diversionary Routing	12.45	14.3	15.55	16.51	17.09	15.18
3	DeLP	4.17	6.45	7.86	9.13	10.09	7.54
